# Genetic divergence and the genetic architecture of complex traits in chromosome substitution strains of mice

**DOI:** 10.1186/1471-2156-13-38

**Published:** 2012-05-18

**Authors:** Sabrina H Spiezio, Toyoyuki Takada, Toshihiko Shiroishi, Joseph H Nadeau

**Affiliations:** 1 Institute for Systems Biology, Seattle, WA, 98019, USA; 2 Mammalian Genetics Laboratory, National Institute of Genetics, Mishima, Shizuoka, 411-8540, Japan; 3 Department of Genetics, Case Western Reserve University, Cleveland, OH, 44106, USA

## Abstract

**Background:**

The genetic architecture of complex traits strongly influences the consequences of inherited mutations, genetic engineering, environmental and genetic perturbations, and natural and artificial selection. But because most studies are under-powered, the picture of complex traits is often incomplete. Chromosome substitution strains (CSSs) are a unique paradigm for these genome surveys because they enable statistically independent, powerful tests for the phenotypic effects of each chromosome on a uniform inbred genetic background. A previous CSS survey in mice and rats revealed many complex trait genes (QTLs), large phenotypic effects, extensive epistasis, as well as systems properties such as strongly directional phenotypic changes and genetically-determined limits on the range of phenotypic variation. However, the unusually close genetic relation between the CSS progenitor strains in that study raised questions about the impact of genetic divergence: would greater divergence between progenitor strains, with the corresponding changes in gene regulation and protein function, lead to significantly more distinctive phenotypic features, or alternatively would epistasis and systems constraints, which are pervasive in CSSs, limit the range of phenotypic variation regardless of the extent of DNA sequence variation?

**Results:**

We analyzed results for an extensive survey of traits in two new panels of CSSs where the donor strains were derived from inbred strains with more distant origins and discovered a strong similarity in genetic and systems properties among the three CSS panels, regardless of divergence time.

**Conclusion:**

Our results argue that DNA sequence differences between host and donor strains did not substantially affect the architecture of complex traits, and suggest instead that strong epistasis buffered the phenotypic effects of genetic divergence, thereby constraining the range of phenotypic variation.

## Background

The phenotypic consequences of inherited mutations and genetic engineering as well as resilience to systems perturbations and response to natural and artificial selection depend heavily on the genetic architecture of complex traits [1]. This architecture includes features such as the number of genes, the nature of DNA sequence differences, the effects of dominance, the extent of epistasis, and the range of pleiotropic actions [2]. But in most cases, we have little genome-wide sense of these features because most studies do not have adequate statistical power. However, new genetic resources, innovations in genotyping, sequencing and phenotyping technologies, and novel analytical methods now enable rigorous studies. Adequately powered studies have recently been reported in yeast, Arabidopsis, cotton, Drosophila, chickens, beetles and other species, with discovery of many complex trait genes (also known as quantitative trait loci, or QTLs) and their interactions [see for example refs. [3-7], but mammalian studies remain especially challenging [8,9].

Chromosome substitution strains (CSSs), also known as consomic strains, provide a special opportunity to characterize the architecture of complex traits in model organisms [10]. CSSs are made by introgressing individual chromosomes from a donor strain onto a host strain background. The first complete CSS panel in mammals was derived by replacing each chromosome in the C57BL/6J (B6) inbred strain with the corresponding chromosome from the A/J strain [11]. Additional mammalian CSS panels have subsequently been made for mice [12] and rats [13]. Although their genetic constitution is highly unusual, CSSs have many unique attributes for gene discovery, functional studies, and systems analysis [14-16]. In particular, by controlling the phenotypic noise of background genetic variation, CSSs have considerable power to identify QTLs and to characterize other genetic features such as epistasis and systems properties such as phenotypic buffering (so-called ‘ceiling and floor’ effects) that are often lost in the background noise of segregating populations [17-23].

With the C57BL/6J-Chr^A/J^ CSS panel, we studied the architecture of complex traits in laboratory mice, focusing on gene number, the nature of their interactions, and related systems properties [14]. This study found at least 466 QTLs among 90 blood, bone and metabolic traits. The picture of genetic architecture was definitive and unexpected. On average, 8 of the 22 CSSs in the mouse panel differed significantly from the host strain for each multigenic trait, with an average phenotypic effect of 76% of the parental difference. Epistasis was detected for 98% of the 41 multigenic traits where the parental strains also differed significantly. The median cumulative phenotypic effect was 803% (range 164% to 1,397%), demonstrating striking non-additivity across these 41 traits. In addition, chromosome substitution led to phenotypic changes that were with few exceptions in the direction of the phenotypic state of the donor strain. Finally, the genetics of the parental strains defined physiological boundaries that largely constrained the range of phenotypic variation. Similar results were found with a rat CSS panel [14], suggesting that these results may be a general feature of inbred strains of mammals, and perhaps of segregating crosses and natural populations, rather than idiosyncrasies of particular traits, strains or species.

Although our study provided new insights about complex traits, many important questions remain, such as the extent to which the close genetic similarity between the progenitor strains affected the results, and whether substituted chromosomes from more distantly related inbred strains would result in more extreme phenotypes and distinct genetic architectures. With increased divergence time, DNA sequence differences accumulate, affecting transcription control and protein functions, and as a consequence phenotypic variation typically increases. But extensive epistasis could buffer sequence differences and constrain the range of phenotypic variation. In the extreme case, genetic background often buffers the phenotypic consequences of loss-of-function mutations [16-19].

Progenitors of mouse CSS panels differ substantially in divergence times. The B6 and A/J strains were derived in the early 1900s from closely related founders among Castle’s mice [24] and have since been inbred for hundreds of generations (jaxmice.jax.org). Although these two strains have many distinct phenotypes (http://www.jax.org/phenome), they are highly similar genetically and their genomes are substantially from the *Mus musculus domesticus* subspecies and many segments of these genomes are identical by descent from a common founder population [25-27]. By contrast, two new CSS panels were recently reported where chromosomes from more distantly related inbred strains were used as a source of donor chromosomes for substitution onto a B6 inbred genetic background [12,28]. PWD/Ph was derived from mice that belong to the *Mus musculus musculus* subspecies and that were trapped in the Czech Republic in 1972 [29]. MSM/Ms belongs to the *Mus musculus molossinus* subspecies and was established from wild mice trapped in Mishima Japan in 1978 [30]. It is estimated that *Mus musculus musculus* and *Mus musculus domesticus* diverged from their common ancestor around 350,000 to 500,000 years ago whereas the divergence time between *Mus musculus molossinus* and *Mus musculus domesticus* is estimated roughly around 1 million years [30-33]. Our study design takes advantage of the introgression of chromosomes from genetically distant strains onto the same host strain background as the C57BL/6J-Chr^A/J^ CSSs.

In this report, we tested whether genetic divergence between host and donor progenitors for CSSs affected the genetic architecture of complex traits. This test was based on comparing a panel of traits for these two new panels of CSSs with results for the original C57BL/6J-Chr^A/J^ panel. The A/J strain diverged from the B6 strain hundreds of generations ago, whereas the progenitors of the PWD and MSM strains diverged from B6 millions of generations ago [24]. As a result, gene regulation and protein functions should differ substantially in the more divergent PWD and MSM strains, both of which show a ~4-fold higher rate of DNA sequence differences than the A/J versus B6 comparison [25-27]. For both panels, we found many large-effect QTLs and pervasive epistasis, as well as strong systems properties that together corroborated results of the original study [14,15]. More importantly, these genetic and phenotypic properties were remarkably similar among the three CSS panels, suggesting that genetic divergence did not have a strong impact on measures of phenotypic variation and that systems properties resulting from pervasive epistasis limited the range of phenotypic variation.

## Methods

### Strains and phenotypes

#### C57BL/6J-Chr^A/J^ CSSs

Results for phenotypic surveys for the C57BL/6J-Chr^A/J^ CSS panel were published previously [11-14] and are used here as a reference for comparison with the results for the PWD and MSM panels.

#### C57BL/6J-Chr^PWD^ CSSs

The panel of C57BL/6J-Chr^PWD/Ph^ CSSs was developed by replacing each B6 chromosome with the corresponding chromosome from the PWD/Ph donor strain. Although the original PWD/Ph panel consisted of 28 strains, only 16 have data presently available in the Mouse Phenome Database (MPD, http://phenome.jax.org/db/q?rtn=projects/projdet&reqprojid=219) [34]. In addition, among these 16 strains, two chromosomes, 11 and X, are each represented with three and two congenic strains because of breeding problems in mice with an intact substituted chromosome. Although absence of a chromosome can result from husbandry issues or from epistasis that compromises fertility or viability, the necessity for two congenic strains rather than a single substituted chromosome is strong evidence for epistasis. In total, 76 traits (38 for males and 38 for females) were downloaded from MPD, including measurements of blood counts and chemistry as well as traits related to bone biology.

#### C57BL/6J-Chr^MSM^ CSSs

The panel of C57BL/6J-Chr^MSM/Ms^ CSSs was made by replacing each C57BL/6J chromosome with the corresponding chromosome of the donor MSM/Ms strain [12]. This panel consists of 28 strains, with chromosomes 2, 6, 7, 12, 13, and X each represented with two congenic strains that either carried the centromeric (C) or telomeric (T) segments of these chromosomes. CSS-10^MSM/Ms^ is absent from the panel because of breeding problems. As with the PWD panel, the need for congenic strains is strong evidence for epistasis.

In total, 71 traits were analyzed. Detailed assay methods and results are published for organ weights, blood chemistry and bone biology traits [12] and available at http://molossinus.lab.nig.ac.jp/phenotype/index.html. Briefly, carcasses were weighed at sacrifice (10 weeks of age) to measure lean body weight. Heart, liver, spleen, kidney and testis were dissected, weighed, and indicated as a percentage of total lean weight. To measure fat pad weight, dorsal and inguinal deposits of subcutaneous white adipose tissues, epididymal, gonadal and mesenteric deposits for visceral white adipose tissues, and inter-scapular and sub-scapular brown adipose deposits were dissected, weighed individually, and indicated as a percentage of total fasting body weight. All animal experiments were approved by the Animal Care and Use Committee of National Institute of Genetics, Mishima, Japan.

For all three CSS panels, care was taken to include a broad range of traits and assays so that phenotyping results were not biased inordinately by idiosyncrasies of particular classes of physiological systems and biological traits. For logistical and historical reasons, similar but not identical assays were surveyed in the three CSS panels [11,12].

### Genetic differences between strains

The patterns of sequence divergence among these strains were obtained from [25-27], (see also http://phenome.jax.org/pub-cgi/phenome/mpdcgi?rtn=snps/Perlegen2_matrix) and were augmented with new sequencing data for the MSM/Ms strain (TT and TS, in preparation). The pairwise polymorphism frequencies were 14% for C57BL/6J and A/J, 57% for C57BL/6J and PWD/Ph, and 54% for C57BL/6J and MSM/Ms. These results are similar to results for more complete sequence analysis of these and other closely related strains [26,27].

### Analytical methods

To be consistent with published studies [14], we focused on traits where at least 3 CSSs differed significantly from the host strain, so that the focus was on multigenic rather than monogenic or digenic control of phenotypic variation. Because most trait measurements were conducted on 8 to 12 mice, with some traits measured in only 4 mice, especially in the C57BL/6J-Chr^PWD/Ph^ CSS panel, we did not use the 3 interquantile range (IQR) to filter outliers (cf. re. 15). Unpaired t-tests were used to determine whether a CSS differed significantly from the host strain after Bonferroni correction for testing multiple strains but not for testing multiple traits.

Because differences in sample sizes and statistical power varied among traits and panels, we examined the frequency distribution of phenotypic effects to test whether significant biases were found among the three CSS panels, but the distributions were similar suggesting that sample size variation did not substantially affect results (Additional file 1: Figure).

Two published methods were used to estimate the magnitude of phenotypic effects [14]. Effect sizes were measured first as a function of the phenotypic difference between the CSS donor and host strains (NormUnit), and second as a proportion of the total phenotypic variance, which was defined by the range of the average phenotypic value among the parental strains and CSSs for each trait (Hi_Low). These methods yielded highly similar results (not shown, but see ref. 17). These alternative measures were needed because the genetic composition of each CSS preclude more conventional estimates of variance and effect size [10,11,14,16].

The directionality of QTL effects was classified into two categories: “moving away from” and “moving towards” trait values for the donor strain (cf. 15). A variable r, which was defined as (T_host_ – T_CSS_)/(T_host_ – T_donor_), was used to demonstrate directionality. When r > 0, the CSS shifted the phenotype “towards” the donor strain, whereas when r < 0, the CSS shifted the phenotype “away from” the donor strain. For r > 1, where the CSS shifted the trait value towards the donor strain, results were also tested to determine whether more extreme trait values than the donor strain were found.

To test for epistasis for each trait, the cumulative signed phenotypic effect was estimated and the standard error propagation method was used to estimate the standard error of measurement (SEM) [14]. We concluded that epistasis dominated phenotypic effects if the absolute value of the cumulative effect minus its SEM exceeded 100%. We note that this somewhat unusual test for non-additivity simply extends across the genome a commonly used method for testing epistasis at pairs of loci [28,29,32-35].

## Results and discussion

### C57BL/6J-Chr^PWD^ CSSs – Mus musculus musculus

We began by analyzing a total of 76 traits in the 16 CSSs in the C57BL/6J-Chr^PWD^ panel. Among the 37 traits that were multigenic, we identified at least 203 QTLs that differed significantly from the C57BL/6J host strain (Table 1). We counted each CSS with a significant effect as having at least 1 QTL affecting the trait of interest, and note that this is a minimum estimate of genetic complexity [15]. We then focused on the 22 multigenic traits where the two parental strains also differed significantly (Table 2). Across these 22 traits, an average of 5.5 CSSs differed significantly from the host strain, at least 122 QTLs were detected and the average phenotypic effect was 153.0% of the difference between the parental strains.

**Table 1:** QTL summary for the three CSS panels

**CSS panel**	**No. of traits**	**Multigenic traits** (n)	**Digenic traits** (n)	**Monogenic traits** (n)	**No. QTLs**	**No. of multigenic QTLs**
A/J	90	62% (55)	10% (9)	12% (11)	16% (14)	437
PWD	76	49% (37)	30% (37)	16% (12)	5% (4)	203
MSM	71	55% (39)	11% (8)	25% (18)	8% (6)	283

For each trait, ‘multigenic’ means at least three CSSs differed significantly from the C57BL/6J host strain, and ‘digenic’ and ‘monogenic’ means two CSSs or only one CSS respectively differed significantly from the host strain, and ‘no QTL’ means no CSSs differed significantly from the host strain. ‘No. of multigenic QTLs’ is the sum of the CSSs with significant effects for all multigenic traits in each CSS panel, assuming that each significant CSS represents at least one QTL on the substituted chromosome.

**Table 2:** QTL number and average phenotypic effect for multigenic traits in the three CSS panels

**CSS panel**	**No. of multigenic traits**	**Ave. no. QTLs per trait** (min, max)	**Ave. phenotypic effect** (%)(min, max)
A/J	41	8.0 (3,16)	75.9 (24.4, 148.4)
PWD	22	5.5 (3,13)	153.0 (29.0, 408.6)
MSM	22	7.2 (3,15)	47.4 (16.6, 148.4)

Ave – average (Min – minimum, Max – maximum). These results apply to traits that showed significant differences for at least three CSSs, and that also differed significantly between the parental strains. The NormUnit method was used to calculate phenotypic effect; the Hi_Low method yielded similar results (not shown).

**Figure 1:**
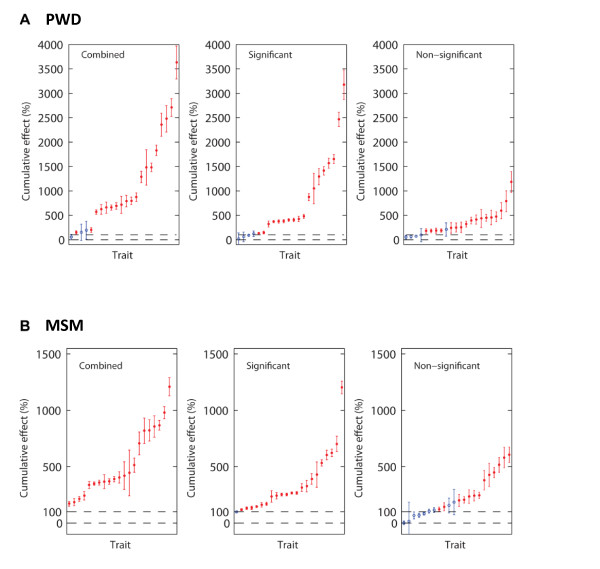
**Cumulative action of combined, significant and non-significant CSS effects for multigenic traits.** For each trait that differed significantly between the parental strains, we calculated the cumulative signed phenotypic effect (summed across the CSSs) and the corresponding SEM. The absolute value of the cumulative phenotypic effect is shown in rank order for each trait. For additive effects, the cumulative phenotypic effect should approach ~100% (dashed horizontal line at 100%). Traits were termed ‘epistatic’ (indicated in red) if the cumulative phenotypic effect exceeded 100% by more than the SEM. The analysis was repeated for only those CSSs whose phenotypic difference from the host strain that achieved statistical significance, those that fell short of statistical significance, and all CSSs combined. **A**. PWD, and **B**. MSM

To test for epistasis, we examined the cumulative signed phenotypic effect and the corresponding standard error of the mean (SEM) for each of these 22 traits. If the cumulative effect minus its SEM exceeded 100%, we concluded that the effects were significantly non-additive [cf. ref. 15]. For each CSS panel, three tests were made, the first focused on CSSs with statistically significant phenotypic effects, the second on CSSs with non-significant effects, and the third on all CSSs combined (Table 3, Figure 1). The rationale for analyzing non-significant effects was based on the observation that any study has limited statistical power and therefore that some true effects may be falsely concluded to be non-significant. If true but statistically non-significant effects occur regularly and in the same direction, their combined effects might in some cases be statistically significant, as was found in a previous study [14]. Among the 22 traits, 82% demonstrated strong epistasis for CSSs with significant effects, with a median cumulative effect of 404.0%. Even for CSSs that did not differ significantly from the host strain, 77% of the traits showed epistasis, with a median of 250.1%. For all CSSs, the cumulative effects exceeded the 100% threshold for 86% of the traits, with a median combined effect of 751.8%.

**Table 3:** Percentage of traits showing non-additive effects (epistasis) and the cumulative phenotypic effects for CSSs with significant effects, non-significant effects, and all CSSs combined

**CSS panel**	**No. of traits**	**Cumulative phenotypic effect**					
		**Significant effects**		**Non-significant effects** (min, max)		**Combined (all) effects**	
		% traits showing epistasis	Med % (min,max)	% traits showing epistasis	Med % (min,max)	% traits showing epistasis	Med % (min,max)
A/J	41	95	605.5 (41.0, 1205.8)	56	168.3 (0.8, 643.4)	98	790.3 (6.5, 1397.2)
PWD	22	82	404.0 (16.8, 3176.9)	77	250.1 (51.4, 1182.5)	86	751.8 (53.2, 3630.8)
MSM	22	91	247.3 (90.7, 1073.7)	50	166.4 (0.3, 523.0)	100	371.7 (156.2, 1589.4)

Med – median (min – minimum, max – maximum).

**Figure 2:**
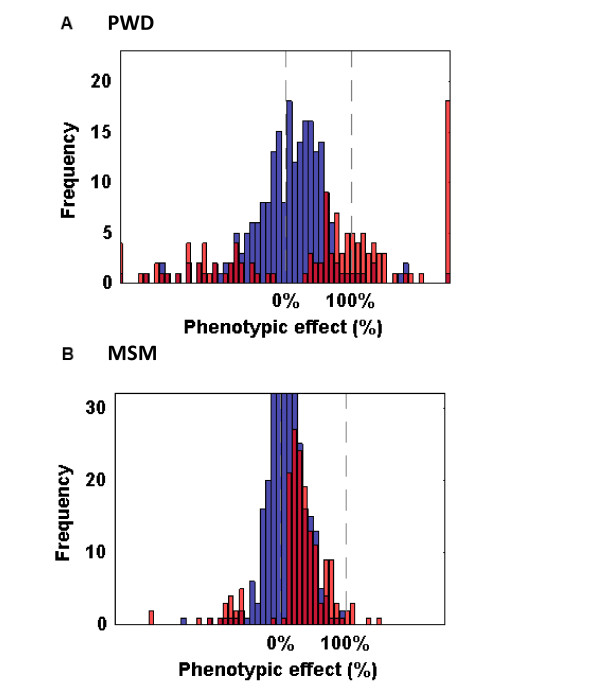
**Frequency distribution of phenotypic effects in two CSS panels.** For traits that differed significantly between the parental strains, phenotypic effects for CSSs that differed significantly from the C57BL/6J host strain are indicated in red, and those that did not differ significantly from B6 are indicated in blue. Phenotypes were normalized so that C57BL/6J = 0% and A/J = 100%. **A**. PWD CSS panel. **B**. MSM CSS panel

Finally, we tested for the directionality of phenotypic effects (Table 4, Figure 2A). Among 122 CSSs that differed significantly from the host strain, 68.9% showed trait values that shifted towards the donor strain and only 31.3% shifted away from the donor strain. For these 22 multigenic traits, 12.3% had more extreme phenotypes than the PWD/Ph donor strain. Directionality of CSSs that did not differ significantly from the host strain also showed a strong shift towards the donor strain (58%, 132 of 226 CSSs). The parental strains defined at least one boundary for the range of trait values for 14 of these 22 traits, and defined both boundaries for 8 traits, implying that the genetics of the parental strains often defined the physiological limits of phenotypic variation for that strain combination.

**Table 4:** Directionality for multigenic traits where the parental strains differed significantly

**CSS panel**	**No. QTLs**	**“away from” donor**	**“towards” donor**	
			**Overall**	**“more extreme“ than donor**
A/J	342	7.3% (25)	92.7% (317)	1.8% (6)
PWD	122	31.3% (38)	68.9% (84)	12.3% (15)
MSM	168	11.9% (20)	88.1% (148)	0% (0)

“Towards donor” and “away from” means that a CSS shifted towards or away from the phenotype of the donor strain. “More extreme than donor” means that a CSS not only shifted the phenotype towards but also significantly beyond phenotype of the donor strain. Number of QTLs is provided in parentheses. The analysis was restricted to multigenic traits where the parental strains also differed significantly.

### C57BL/6J-Chr^MSM^ CSSs – Mus musculus molossinus

Among the 71 traits that were surveyed for the 28 CSSs in the C57BL/6J-Chr^MSM/Ms^ panel, 39 were multigenic, and a total of 283 QTLs differed significantly from the host strain (Table 1). We then focused on the 22 traits where the parental MSM/Ms and C57BL/6J strains also differed significantly (Table 2). For these traits, an average of 7.2 CSSs differed significantly from C57BL/6J, at least 163 QTLs were discovered, and the average phenotypic effect of these CSSs was 47.4% of the parental difference.

We next examined the cumulative effects for these traits across the C57BL/6J-Chr^MSM/Ms^ CSS panel (Table 3, Figure 1B). Among the 22 traits, 91% demonstrated strong epistasis for CSSs with significant effects, with a median cumulative effect of 247.3% (range 117.6% to 1,073.7%), for CSSs with non-significant effects, 50% traits showed epistasis, with a median cumulative effect of 166.4%, and for all CSSs, 100% (22) of the traits showed non-additive effects, with a median cumulative effect of 371.7%.

Lastly, we focused on directionality of phenotypic effects (Table 4, Figure 2B). Among the 163 CSSs with statistically significant effects, 88.1% shifted the phenotype ‘towards’ the MSM/Ms donor strain and only 11.9% shifted ‘away from’ the donor strain. Even for those non-significant CSSs, 70% (311 of 447) shifted ‘towards’ and 30% (136 of 447) shifted ‘away from’ the donor strain, again indicating a strong bias towards the donor strain. None of the CSSs had trait values that were more extreme than the donor strain. The parental strains defined both boundaries for 17 (77%) of the 22 traits, and one boundary for 5 traits.

### Effects of genetic divergence on phenotypic variation

Finally, we tested whether more divergent strain combinations showed more distinctive phenotypic and systems features. If genetic divergence is critical, then the MSM and PWD CSSs should differ more than the A/J CSSs from the B6 reference strain, whereas if epistasis and systems properties constrained the range of phenotypic variation, measures of phenotypic variation should be similar among the three CSS panels. To test this hypothesis, lineage-specific frequencies of DNA sequence variation [25-27] were used as a measure of genetic divergence for comparisons with the various measures of phenotypic variation in each of the three CSS panels (Table 5). In particular, the 4-fold difference in frequency of DNA polymorphisms for among the three donor strains relative to the C57BL/6J reference strain suggests that divergence of non-coding regulatory elements as well as protein functions should also differ substantially in these strain comparisons [25-27]. We also note that the time since divergence differs substantially among the progenitor strains of the CSS panels, i.e. 100 years versus 350,000 - 1,000,000 years [24-27,30-33].

**Table 5:** Features of genetic architecture and systems properties in the three CSS panels

**CSS panel**	**Percentage of traits that are multigenic**	**Ave. no. of QTLs per trait**	**Ave. phenotypic effect** (%)	**Percentage of traits showing epistasis**	**Median cum. effect**	**“away” from donor**
A/J	62%	8.0	75.9	98%	790.3%	7.3%
PWD	49%	5.6	152.9^1^	86%	751.8%	31.3%^3^
MSM	57%	7.7	47.2^2^	100%	412.0%	11.9%

Each of the three CSS panels was compared to the C57BL/6J host strain. Various features of genetic and systems architecture of complex traits were compared for the two CSS contrasts, namely CSS_PWD_ vs CSS_A/J_ and CSS_MSM_ vs CSS_A/J_. Statistical tests were applied to average phenotypic effect (%), percentage of traits showing epistasis, median cumulative effect, and phenotypic shift ‘away from donor’ strain, but were not used for percentage of traits that are multigenic and average number of QTLs per trait, because both the PWD and MSM panels are incomplete (missing CSSs).

^1^ *t*-test: P < 10.^-20^

^2^ *t*-test: P < 10.^-16^

^3^ χ^2^ contingency test: P < 0.001.

Interestingly, several lines of evidence suggest that CSSs that were derived from more divergent strain combinations were not consistently more extreme than those from more recently derived progenitors (Table 5). First, the average number of CSSs that differed from the host strain across the panel of traits was similar for A/J and MSM, and both were somewhat greater than the number for PWD. Second, the percentage of traits showing epistasis was similar among the three CSS panels (range 86% to 100%). Third, the average phenotypic effect was significantly greater for A/J versus B6 than for MSM versus B6, but significantly less than PWD versus B6, i.e. PWD > A/J > MSM. Fourth, the median cumulative phenotypic effect was similar for A/J versus B6 and PWD versus B6, and both were roughly twice the effect for MSM versus B6, i.e. A/J = PWD > MSM. Finally, the phenotypic shift “away from donor” was significantly stronger for PWD versus B6 than for the two other CSS versus B6 comparisons, i.e. PWD > A/J = MSM. Thus we found no consistent evidence that genetic divergence systematically affected genetic architecture or systems properties.

The incomplete nature of the PWD and MSM CSS panels has implications for several measures of genetic architecture and systems properties. In particular, the number of QTLs as well as their cumulative phenotypic effect may be under-estimated because the missing CSSs may carry QTLs affecting traits of interest, whereas estimates of average phenotypic effect size as well as trends in systems properties such as directionality or boundaries of phenotypic effects should not be affected. If the four missing strains from the PWD panel and the one strain missing from the MSM panel were similar to those that were studied, we estimate 6.7 QTLs (=5.5 x 22/18) for PWD and 8.1 QTLs (= 7.2 x 22/21) for MSM respectively for CSSs that have at least one QTL with a significant phenotypic effect. Thus complete panels would probably have provided even more consistent results across the three CSS panels. Similarly, pairs of congenic strains rather than a complete substituted chromosome should not significantly affect the results because they maintain the CSS paradigm of single large genome segments isolated on a defined genetic background.

Finally, we note that while ‘ceiling-floor’ effects might constrain the range of phenotypic variation for individual traits, these effects have negligible impact on measures of phenotypic variation across the CSS panels. Indeed, the ‘percentage of traits’ was the only measure that was ‘saturated’ with all three panels approaching 100%; the four other measures could have yielded more extreme results, i.e. additional ‘CSSs with significant effects’ could have been discovered, and the ‘average effect size’, the ‘median cumulative phenotypic effect’, and the ‘phenotypic shift’ could each have been greater.

Although theoretical aspects of the relation between functional differences and genetic divergence have not been studied extensively, the reasonable expectation is that increasing genetic divergence should lead to more dramatic phenotypic and systems differences. The present study not only corroborated results of the original CSS survey [14,36], but also showed that differences in regulatory elements and protein functions related to genetic divergence between progenitor strains did not lead to more extreme phenotypic variation. We analyzed CSS panels that were derived from two genetically distant *Mus musculus* subspecies, *Mus musculus musculus* (PWD/Ph; and *Mus musculus molossinus* (MSM/Ms; [12]), respectively. Genotyping surveys as well as complete genome sequencing reveals a ~4-fold higher rate of DNA sequence variants between the more divergent strains (PWD and MSM versus B6) as compared to the more closely related strains (A/J versus B6) [25-27]. Surveys of a broad variety of traits in all three panels revealed large phenotypic effects and pervasive epistasis for the majority of traits. Overall, the phenotypic features for CSSs derived from these more genetically divergent MSM and PWD inbred strains were remarkably similar to those derived from the A/J inbred strain (Table 5).

To account for phenotypic features that are largely independent of the extent of genetic divergence, we propose that organisms are composed of non-random combinations of allelic variants that provide not only the necessary conditions for viability, fertility and other essential aspects of organismal biology, but resilience to environmental and genetic perturbations. Under these conditions, genetic divergence is less important than favorable combinations of genetic variants. Studies of spontaneous, induced and engineered mutations, modifier genes, and genetic background effects support the hypothesis that epistasis buffers organisms from the adverse phenotypic effects of deleterious mutations and naturally-occurring genetic variants [17-19].

Issues that emerge from this work include developing a theory that accounts for the ways that functional differences scale with genetic divergence and obtaining experimental evidence for the molecular and systems properties that buffer organisms from environmental and genetic perturbations.

## Conclusions

In this study, we tested whether genetic divergence leads to more distinct phenotypes, or alternatively whether epistasis constrains patterns of phenotypic variation. The expectation was that various measures of phenotypic differences and several systems properties would be quite distinct in that the new CSS panels have 4-fold difference in DNA sequence divergence versus the original panel. We compared patterns of phenotypic variation among three panels of chromosome substitution strains (CSSs) of mice, and the remarkably similar results across the panels suggest that epistasis, which is a pervasive feature in these strains, constrained the patterns of phenotypic variation. These results contribute to the growing body of evidence that biological systems tend to be composed predominately of epistatic networks of genes action rather than collections of genetic variants with independent and additive effects.

## Authors’ contributions

SHS and JHN designed the study and wrote the paper. SHS performed the data analysis. TT and TS provided phenotypic data and SNP information of MSM panel. All authors read and approved the final manuscript.

## Supplementary Material

Additional file 1 Figure 1 Frequency distribution of phenotypic effect sizes for the three CSS panels, based on the Hi_Low method – the NormUnit method yielded similar results (not shown). Click here for file
