# Supplementary figures and images for: Genetic divergence and the genetic architecture of complex traits in chromosome substitution strains of mice
Source: BMC Genet. 2012 May 18;13:38. doi: 10.1186/1471-2156-13-38 (PMC3406986; doi:10.1186/1471-2156-13-38)

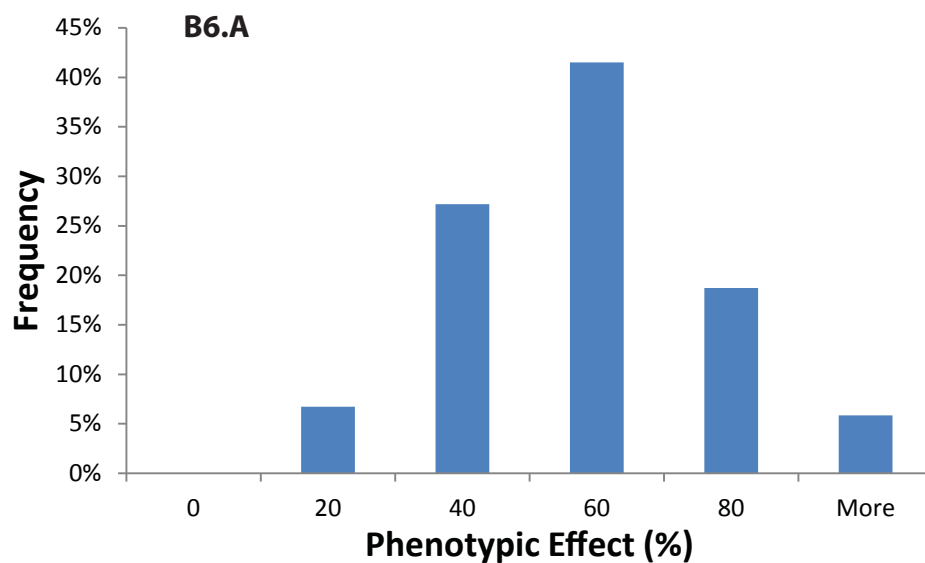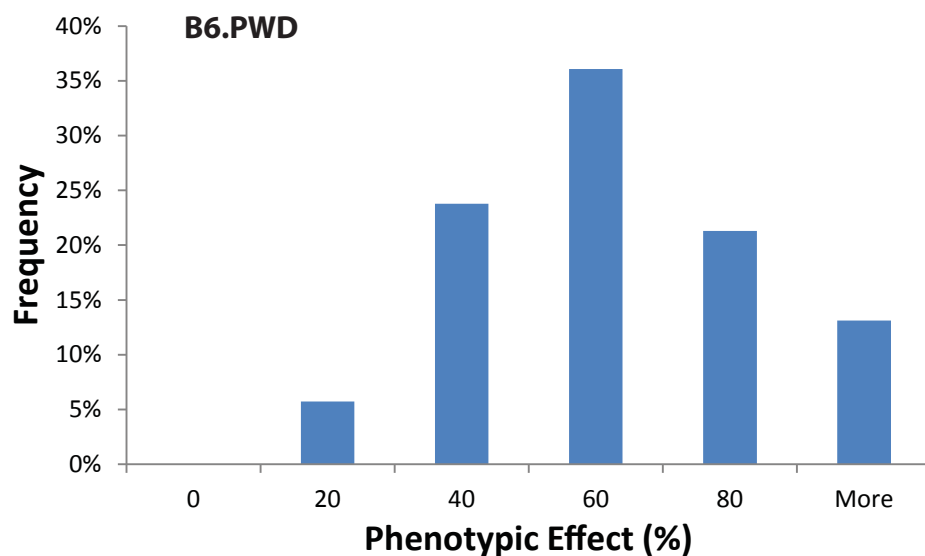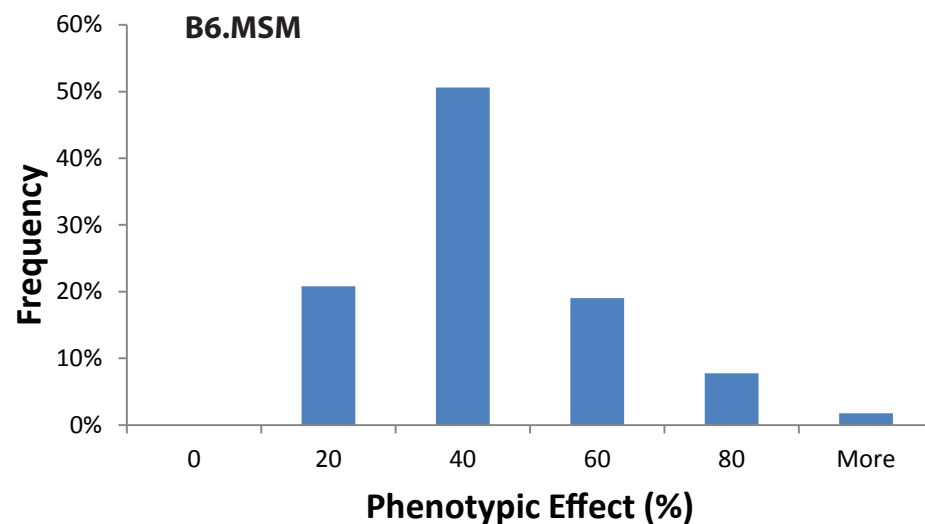

Supplement: Additional file 1 — Figure 1 Frequency distribution of phenotypic effect sizes for the three CSS panels, based on the Hi_Low method – the NormUnit method yielded similar results (not shown). [file 1471-2156-13-38-S1.pdf]
